# Exploring the burden of X-linked hypophosphatemia: a European multi-country qualitative study

**DOI:** 10.1007/s11136-020-02465-x

**Published:** 2020-03-11

**Authors:** S. H. Lo, R. Lachmann, A. Williams, N. Piglowska, A. J. Lloyd

**Affiliations:** 1 Acaster Lloyd Consulting Ltd, London, United Kingdom; 2 University College London Hospitals, London, United Kingdom; 3 Kyowa Kirin Services Ltd, Marlow, United Kingdom

**Keywords:** X-linked hypophosphatemia (XLH), Metabolic bone disease, Rare hereditary disease, Burden of disease, Quality of life, Pain, Stiffness, Fatigue

## Abstract

**Introduction:**

X-linked hypophosphatemia (XLH) is a rare, lifelong, progressive disease characterised by renal phosphate wasting and abnormal bone mineralisation. Symptoms begin in early childhood, with the development of rickets and related skeletal deformities and reduced growth, progressing to long-term complications, including pseudofractures and fractures, as well as pain, stiffness and fatigue. The present study was designed to explore the patient experience of pain, stiffness and fatigue and the psychosocial impact of XLH in detail.

**Methods:**

A cross-sectional qualitative study was conducted in the United Kingdom (18), Finland (6), France (4), Germany (1) and Luxembourg (1) with XLH patients aged 26 and over. Interview discussion guides were developed in consultation with clinical experts and patient associations. Data were analysed thematically.

**Results:**

Participants (*N* = 30) described pain, stiffness and fatigue as frequently experienced symptoms with a significant impact on physical functioning and activities of daily living (ADLs). Some also described the symptoms as impacting their mood/mental health, relationships, social life and leisure activities. Participants described how common symptoms could interact or aggravate other symptoms. Symptoms had often worsened over time, and for many, were associated with concern about the future. Most participants were worried or felt guilty about having children with XLH. The findings confirmed and extended the existing model of the burden of XLH.

**Conclusion:**

The present study is the first to provide an in-depth analysis of pain, stiffness and fatigue, their impact and the interrelatedness of these symptoms among adults with XLH. The study also described the psychosocial impact of XLH as a hereditary, lifelong progressive disease.

**Electronic supplementary material:**

The online version of this article (10.1007/s11136-020-02465-x) contains supplementary material, which is available to authorized users.

## Introduction

X-linked hypophosphatemia (XLH) is a rare, lifelong, progressive disease caused by a defect in the PHEX gene, leading to excessive levels of circulating FGF23 with a prevalence rate of around 1 in 20,000 [1, 2]. This leads to hypophosphatemia and resultant defective bone and tooth mineralisation. The condition first emerges in childhood, manifesting as vitamin D-resistant rickets, with bowed legs, other skeletal deformities and short stature, leading to impaired physical functioning and pain [3, 4]. The chronic nature of XLH leads to ongoing bone and joint damage, softening of the bones and reduced mobility. Long-term complications, including but not limited (pseudo-)fractures and osteoarthritis can occur [5, 6]. Dental issues are common, including frequent abscesses and tooth loss, often requiring orthodontic intervention [7, 8].

For the past forty years, children and adults with XLH have received conventional therapy, consisting of oral phosphate supplementation given in multiple daily doses and active vitamin D analogues. Despite this treatment, XLH can be associated with incomplete healing of rickets, residual skeletal deformity, and persistent short stature. Furthermore, the treatment with phosphate is associated with gastrointestinal side effects and the risk of metabolic and endocrine abnormalities such as hypercalciuria, nephrocalcinosis and hyperparathyroidism. In addition, skeletal abnormalities and dental anomalies acquired in childhood often require surgical correction at the end of growth.

Relatively few studies on health-related quality of life (HRQoL) in XLH patient populations have been published to date. The evidence suggests that the XLH HRQoL is comparable with other rare musculoskeletal diseases such as osteogenesis imperfecta and fibrous dysplasia and higher than that of similar, but more common musculoskeletal disorders such as axial spondyloarthritis [4, 9]. Relatedly, a recent survey among adults with XLH showed low satisfaction with HRQoL and average physical function scores of more than one standard deviation below the population norm [10].

A recent qualitative study presenting an overview of the adult XLH patient experience highlighting the disease’s multi-faceted symptomatology and varied impacts on patients’ lives [11]. The study identified pain, stiffness and fatigue as very common symptoms among adults with XLH and highlighted impacts on ADLs, physical functioning, emotional well-being, sleep and the need for long-term adjustments such as assistive devices and modifications to patients’ homes. The study was conducted in a US-based English-speaking sample of 15 women and 3 men. The authors present initial findings regarding pain, stiffness and fatigue, but did not provide an in-depth analysis of the experience of these symptoms in XLH and their impact on physical, mental and social well-being as well as ADLs. The emotional and psychosocial impact of living with XLH as a lifelong hereditary condition has not been explored.

The primary objective of the present study was to provide an in-depth, qualitative understanding of the nature and impact of pain, stiffness and fatigue symptoms in XLH as well as the psychosocial impact of XLH as a lifelong hereditary condition. We further assessed whether the XLH patient experience as described by a more heterogenous sample in terms of age, gender and disease severity across several European countries was consistent with a previous patient experience study by Theodore-Oklota and colleagues [11].

## Methods

### Study design

A cross-sectional qualitative study was conducted with adult XLH patients living in Finland, France, Germany, Luxembourg and the United Kingdom between January and April 2019. A background questionnaire (Supplementary material 1) and interview discussion guide (Supplementary material 2) for semi-structured telephone interviews were developed using published literature on the health-related quality of life of XLH patients and results of a rapid review of published instruments used to measure pain, stiffness and fatigue. The semi-structured interview consisted pre-dominantly of open-ended questions regarding patients’ medical history, symptoms, their daily lives and how XLH impacts their lives, current treatment and care, followed by more in-depth questions on any reported pain, stiffness and fatigue symptoms. The rapid review was used to identify appropriate follow-up questions and probes on the subjective experience, severity, frequency, duration, patterns and impacts of these symptoms. Clinical experts and patient advocacy groups were subsequently consulted to provide feedback during the study design process. All English language study materials were translated into Finnish, French and German. The study protocol was reviewed and approved by the WIRB-Copernicus Group Independent Review Board (IRB tracking number: 20183122). Translated study materials were certified and also submitted for IRB approval. All translations for this study were provided by professional translators.

### Sample

Study participants were recruited through patient associations across Europe using short recruitment advertisements via social media or emails. Eight patient associations were contacted with a request for help with patient recruitment; of these, four agreed to help. All participants who expressed an interest in the study were asked a few brief screening questions via email to ascertain whether they met the inclusion criteria of (i) having a (self-reported/not independently verified) clinical diagnosis of XLH; (ii) never having received burosumab (Crysvita) as a treatment for XLH; and (iii) aged 26 years or over. Patients on burosumab were excluded to ensure the patient experience on conventional therapy was captured; too few patients would have been on the newly licenced treatment for long enough to be able to compare and contrast subgroups. Patients aged under 26 were excluded to enable us to examine the patient experience of XLH in adulthood in a sample of fully grown adults who are not transitioning from one phase of their life to another. Some men continue to grow into their early 20s and age 25 is a conventional cut-off point to distinguish between young adults and adults. Once eligibility was confirmed, participants were asked via email to provide their consent to proceed with the interview in the local language.

### Interview procedures

All interviews were conducted over the telephone by experienced qualitative interviewers who were briefed by the first author (except interviews conducted by the first author) on the study objectives in a 1-h interviewer briefing. Interviewers first provided a brief explanation of the purpose of the study, the interview process and asked participants to re-confirm their consent. The interview started with questions from the background questionnaire, followed by a semi-structured interview guided by the discussion guide. Telephone interviews lasted approximately one hour. All English language interviews were recorded and transcribed verbatim. Interviews conducted in other languages were recorded, simultaneously translated and transcribed the translated text verbatim into English.

### Analysis

Data from the background questionnaire were summarised. Semi-structured interview data were used to supplement background questionnaire responses if initial responses appeared incomplete. Interview transcripts were coded and analysed using thematic analysis [12, 13]. Transcripts were coded and analysed using qualitative research software (MAXQDA). All transcripts were coded by one researcher and a second researcher independently coded 10% of transcripts. Researchers reviewed and compared codes to ensure there were no major discrepancies in interpretation of the data meaning that the coders did not disagree on the meaning of the text and codes. A coding framework was developed iteratively throughout the analysis (final version in Supplementary material 3). Codes were initially derived from concepts covered in the discussion guide. The initial coding framework was then revised to remove ambiguity in codes and supplemented with codes that covered concepts that emerged from the analysis. The aim of the analysis was to identify relevant concepts, which were subsequently grouped into themes pertinent to the patient experience of living with XLH. Saturation matrices were created to summarise the frequency of code application across all interviews. The matrices suggest that data saturation was reached: no new codes were applied after sixteen interviews with each code having been applied at least twice.

## Results

### Sample characteristics

A total of 30 participants living in the United Kingdom (18), Finland (6), France (4), Luxembourg (1) and Germany (1) took part in the telephone interviews. The sample demographic characteristics and clinical characteristics are summarised in Tables 1, 2, respectively.

**Table 1:** Sample demographic characteristics of XLH adults aged 26 and over (*N* = 30)

	Mean	Range
Age	40	26–69

	*N*
Sex	
Male	9
Female	21
Employment status	
Employed, full-time	19
Employed, part-time	3
Unable to work due to health	4
Unemployed/volunteer	3
Retired	1
Living situation	
Living with partner	26
Living alone	2
Living with family	2
Educational level	
No formal qualifications	1
Secondary education	9
Professional qualification	6
Degree level or above	14

**Table 2:** Sample clinical characteristics of XLH adults aged 26 and over (*N*= 30)

	Mean	Range
Age at diagnosis	9	0–47

	*N*
XLH severity (self-reported)	
Mild	7
Moderate	10
Severe	9
Don't know	4
Current treatments	
None	*5*
Phosphate	22
Calcitriol/Vitamin D	24
Dental procedures	7
Surgery	6
Physiotherapy	13
Analgesicsa	22
Other treatments	9
Past treatments	
Phosphate	28
Calcitriol/Vitamin D	29
Dental procedures	27
Surgery	22
Physiotherapy	21
Growth hormone	2
Analgesics^a^	2
Other treatments	5
Family history (mother or father)	
Yes	16
No^b^	14
Co-morbidities	
None	17
Musculoskeletal co-morbidities	7
Other co-morbidities	8

^a^Not a prompted response option on the background questionnaire

^b^Reasons included having a parent with no confirmed diagnosis of XLH, being adopted and having a spontaneous gene mutation

### Symptom overview

Pain, stiffness, fatigue and mobility issues were the most common symptoms of XLH that participants reported as affecting their lives, with at least nine in ten reporting these symptoms. However, unlike pain and stiffness symptoms, many participants were unsure if their fatigue fell within the range experienced by healthy people without XLH or suspected that their fatigue could have causes other than XLH. Only a few participants attributed the fatigue they experienced (primarily) to XLH.

Dental issues, in particular dental abscesses, and short stature were underlying musculoskeletal characteristics of XLH reported by more than three-quarters of the sample. A majority reported bone bowing and other skeletal deformities, but for some these had been corrected through surgery. Some also reported balance issues, fractures or soft/weak bones, and hearing loss or tinnitus. Only one participant reported no current symptoms or underlying musculoskeletal features of XLH (e.g. short stature).

Participants often expressed ambivalence towards the effectiveness of conventional XLH treatment due to a perceived lack of or limited impact on XLH symptoms.

### Overall impact of XLH: overview

Commonly reported physical impairments included difficulty walking, walking slower than average, difficulty standing for long and difficulties with movements requiring joint flexibility. Participants described the negative impact of XLH on their ability to do housework (e.g. standing for long to cook, grocery shopping); caregiving (e.g. carrying children, walking quickly or for long with children) and any social, leisure or work activities that required prolonged periods of walking or standing. Many were unable to run or take part in any high-impact sports. Some participants reported using assistive devices or having modified living and working environments to enable them to live with mobility issues and short stature.

The majority of participants reported some psychosocial impact of XLH. Negative social experiences were very common and included bullying, being stared at and other unkind behaviours in response to their physical appearance. Participants also talked about unwanted attention due to the need to take medication in public. These experiences had often affected participants’ psychological well-being, often resulting in low self-esteem, feelings of being ‘different’ from other people, frustration and depression. Many also reported going out less frequently as a result of XLH. Nevertheless, it was also common for participants to report having developed mental resilience as a consequence of XLH which they perceived as a positive impact and helped them cope with their condition.

### Self-reported severity

Participants were asked how severe they perceived their XLH to be; no guidelines were given to define mild, moderate and severe. Those who self-reported mild XLH tended to describe a better overall quality of life and less severe symptoms than those who perceived their XLH as moderate/severe. Two participants who had no pain, stiffness or skeletal deformities described their XLH as mild. The self-reported mild participants stated that they were able to work, whilst some moderate/severe patients were unable to work due to ill health. Self-reported moderate/severe participants were more likely to report the use of assistive devices and moderate to severe pain, stiffness and/or fatigue than those participants who considered their disease to be mild.

### XLH patient experience of pain

**Fig. 1:**
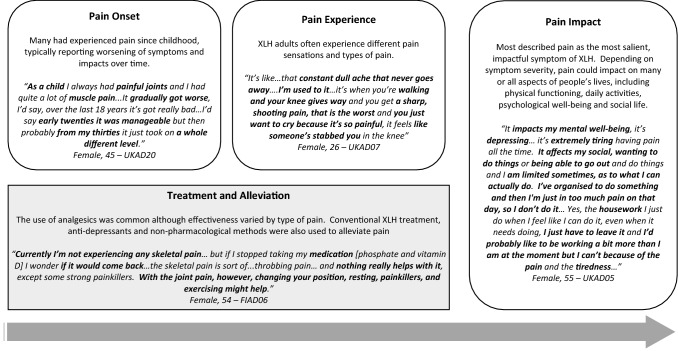
Overview of the XLH patient experience and impact of pain with illustrative quotes

The experience and different impacts of pain are summarised alongside illustrative quotes in Fig. 1. Most participants described more than one type of pain, indicating that pain is a multi-faceted symptom of XLH. Adults with XLH often reported a combination of joint, bone and/or muscle pain as well as both chronic and acute pain. A wide range of adjectives were used to describe pain sensations including ‘dull’, ‘achy’, ‘twinging’, ‘throbbing’, ‘sharp’, ‘stabbing’ and ‘electric’. Different types of pain would often be reported by the same participant.

Many participants experienced pain on a daily basis. The severity of pain fluctuated but most reported a constant base level of pain even during milder periods. Pain severity ranged from mild to extremely severe. Pain severity typically varied from day to day. The duration of more severe and ‘acute’ pain episodes typically varied widely, ranging from minutes to weeks, depending on the type of pain.

### Onset, changes over time and triggers of pain

Many participants had experienced pain since childhood. Some recalled early adulthood as a period least impacted by pain. Others reported the onset of pain in adulthood, mostly in their mid- to late twenties and thirties. Among those who reported a change in pain over time, this was usually a worsening of pain. A few reported less pain following successful surgery.

Movement was most frequently described as a pain trigger, including walking, standing and sitting. Quick movements or specific movements or postures were also common triggers. Other salient pain triggers included weather and season-related factors, typically cold and/or humidity. If fluctuations were experienced over the course of the day, pain tended to be most severe in the mornings and/or at night. People also reported that being stationary could trigger pain.

### Pain medication and alleviation

Most participants used analgesics to cope with pain. A wide variety of pain medication was used, ranging from over-the-counter painkillers to steroid injections and morphine. Treatment effectiveness was perceived as variable and participants often reported that painkillers reduced the pain but did not relieve it completely. Painkillers were sometimes perceived to have varying levels of effectiveness on different body parts. Some people also used anti-depressants and conventional XLH treatment to reduce pain symptoms. Furthermore, a wide range of non-pharmacological methods were used. These included hot water (baths, hot water bottles), exercise and stretching, physiotherapy, massage, and alternative therapy (e.g. acupuncture).

### Impact of pain on daily activities and psychological well-being

Pain typically impacted on many or all aspects of people’s lives, depending on symptom severity. For many, pain was the most salient symptom of XLH, leading to difficulties walking/running, standing, sitting for long or specific types of movements. Specific movements that were characteristically affected included bending knees, lifting things and quick movements. Mobility problems, in turn, affected daily activities, such as work, housework and shopping, and sometimes, also self-care due to difficulties with specific movements requiring joint flexibility (e.g. getting dressed, bathing). Parents often described the impact of pain on caregiving and their ability to play with their children, and some cited this as one of the most challenging aspects of XLH. Some participants also reported an impact of pain on sleep, for example, because of difficulties finding a comfortable sleeping position.

Pain had a considerable psychological impact, with people commonly describing it as affecting their mood (e.g. feeling down or depressed) depending on pain severity. Pain also impacted participants’ social life, as it often affected motivation to go out or led to plans being cancelled. Some also described active social pursuits (e.g. walking, sports) that they could not take part in as a consequence of their pain.

### XLH patient experience of stiffness

**Fig. 2:**
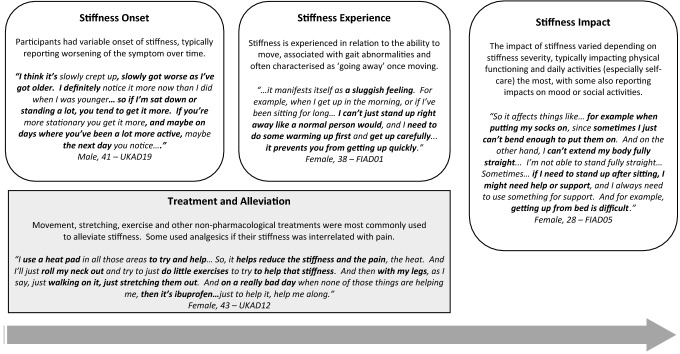
Overview of the XLH patient experience and impact of stiffness with illustrative quotes

The patient experience of stiffness and its impacts are summarised alongside illustrative quotes in Fig. 2. Stiffness was generally described as restricting or affecting movement. Words to describe stiffness included ‘locking’, ‘heavy’, ‘sluggish’, ‘stuck’, ‘clicking’, ‘frozen’, ‘rusty’ and ‘restricted’. Stiffness was linked to the ability to move, either to start movement or to enable specific movements. Some participants described their gait as walking like a ‘duck’, ‘robot’ or ‘penguin’.

A wide range of stiffness severity was reported, ranging from mild to extremely severe, with some experiencing a constant level of stiffness while others had large fluctuations in severity. Most people experienced stiffness every day, although some reported only experiencing it on a weekly basis.

### Onset, changes over time and triggers of stiffness

The age of onset of stiffness symptoms was variable, ranging from childhood to middle age. A few had noticed the onset of stiffness following surgery. Participants commonly noticed a worsening of stiffness over time, although a few felt symptoms had been stable or had improved. Estimates of symptom onset and changes over time were perceived as imprecise, as some suggested it was difficult to be precise due to the gradual nature of changes in stiffness.

Stiffness was characteristically experienced after being in the same position for a long time. Less-frequently reported triggers were similar to those mentioned for pain, including exercise and weather-related factors (cold, humidity). Most reported worse stiffness in the mornings. A minority experienced stiffness both in the mornings and evenings, solely in the evenings or perceived no pattern in the time of day. In contrast to pain, participants usually described stiffness as ‘going away’ once they started moving. Only a few experienced longer episodes of stiffness.

### Stiffness treatment and alleviation

Most participants described using movement as a tool to overcome their stiffness. They would typically ‘move’, ‘exercise’ or ‘stretch’ to relieve stiffness. Specific exercise used to alleviate stiffness included walking, jogging, yoga, Pilates and water-based exercise. Heat (hot water, hot oil, clothes) was also commonly used to relieve stiffness. Physiotherapy, massage, acupuncture and chiropractic were also used to alleviate stiffness, often in conjunction with treating any pain participants had. Some also mentioned using painkillers, typically when they perceived stiffness and pain as inter-related. A few participants reported that they had no means to alleviate their stiffness.

### Impact of stiffness on ADLs and psychological well-being

Stiffness impacted on many aspects of people’s lives, depending on its severity. Although pain was the dominant symptom for most, a few reported that stiffness was the most impactful symptom for them. Mobility-specific impacts were related to difficulties with starting movement or specific movements that required the bending of joints. This affected getting in and out of bed, chairs or cars, going up and down stairs or movements such as squatting and bending forward. Gait abnormalities due to stiffness also lead to some needing to ‘shuffle’, ‘hobble’, ‘limp’ and use assistive devices when walking. Mobility-related impacts affected daily activities, including self-care (getting dressed, bathing), housework and work due to difficulties bending joints. Similar to pain, parents also reported stiffness-related impacts on caregiving and their ability to play with their children. Some also reported stiffness as affecting mood, resulting in feelings of frustration. Only a few reported impacts on social and leisure activities.

### XLH patient experience of fatigue

**Fig. 3:**
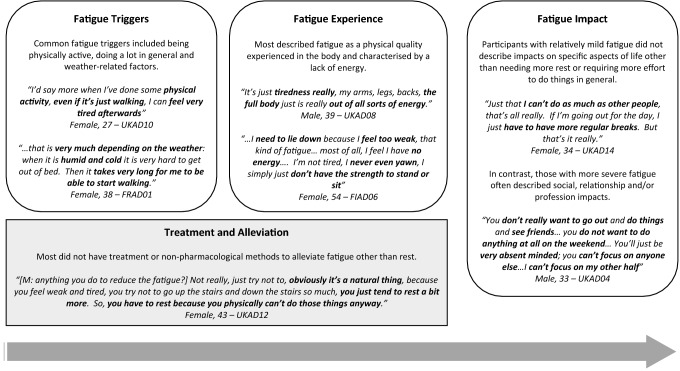
Overview of the XLH patient experience and impact of fatigue with illustrative quotes

The patient experience of fatigue and its impacts are summarised alongside illustrative quotes in Fig. 3. Most participants described fatigue as having a physical quality that is experienced in the body and characterised by a lack of energy. Some also associated it with muscle fatigue or weakness. Words used to describe fatigue included ‘tired’, ‘sluggish’ and ‘worn out’. Some also noted that their fatigue was not relieved by rest or sleep. A few also reported needing to sleep during the day as a result of fatigue.

Fatigue severity ranged from very mild to severe, with most participants reporting moderate levels of fatigue. The frequency of fatigue symptoms was variable, with only a minority experiencing daily fatigue. Most reported increased fatigue in the evenings, although a few also experienced being more fatigued in the mornings.

### Onset, changes over time and triggers fatigue

Fatigue onset was extremely variable, with some having experienced fatigue since childhood while others did not experience it until they were middle aged. A few linked the onset of fatigue to a post-surgery recovery period. A worsening of fatigue symptoms over time was only reported by a small minority.

The most commonly reported triggers were being physically active and doing a lot in general. A few also reported weather-related factors (cold, humidity) as fatigue triggers. Pain, and sometimes associated sleep problems, were also reported to contribute to fatigue.

### Treatments and alleviation of fatigue

Most participants did not report using treatments or strategies to reduce fatigue other than resting, taking breaks and sleeping. A few participants mentioned eating well, staying active and muscle strengthening exercises to aid sleep in the context of alleviating fatigue.

### Impact of fatigue on ADLs and psychological well-being

Varying impacts stemming from fatigue where reported, depending on fatigue severity and participants’ personal circumstances. For some, impacts were non-specific and described as needing more rest, time or effort to do things. However, others reported major impacts on key areas of daily life such as work, caregiving, relationships and social life. Work-related impacts included early retirement, a desire to reduce working hours, sick leave, arriving late at work and loss of concentration during work time. Participants with children sometimes reported doing fewer caregiving activities or relying more on their partners for caregiving duties.

### Interrelatedness of pain, stiffness and fatigue

**Fig. 4:**
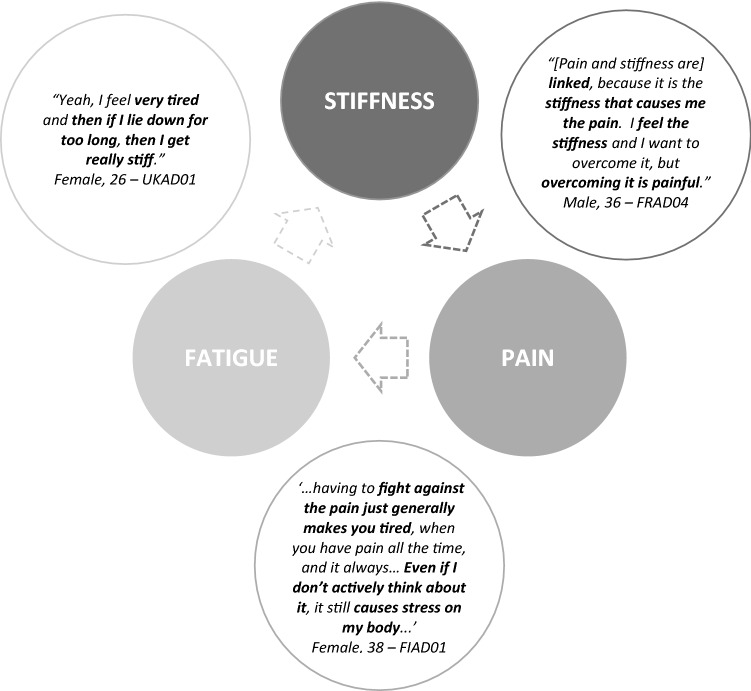
Model of reported links between pain, stiffness and fatigue symptoms in XLH with illustrative quotes

Some participants perceived stiffness, pain and fatigue as interlinked in a mutually aggravating cycle as shown in Fig. 4. Most described pain in conjunction with stiffness, with the two as distinct though often co-occurring symptoms. Stiffness tended to be viewed as causing or aggravating the pain. The association between pain and fatigue was less consistent; however, some noted that pain made fatigue worse, sometimes due to the impact of pain on sleep. A few also noted that resting to relieve fatigue aggravated their stiffness.

### The psychosocial impact of XLH as a lifelong, hereditary, progressive condition

**Fig. 5:**
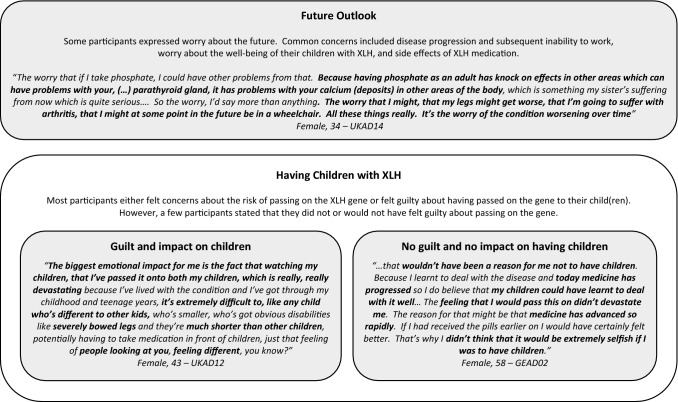
Overview of the psychosocial impact of XLH as a lifelong hereditary condition with illustrative quotes

The psychosocial impact of XLH as a lifelong, hereditary condition are summarised together with illustrative quotes in Fig. 5. Some participants expressed worry about the future. The most common concerns included disease progression and subsequent inability to work, worry about the well-being of their children with XLH, and side effects of XLH medication. Many who had experienced a deterioration in their health over time were concerned that symptoms, especially pain and stiffness, would get worse with age, leading to worse physical functioning and further operations. A few also reported observing their elderly parents’ condition worsening over time which caused concern for their own future.

The hereditary nature of XLH, and specifically its impact on having children, was discussed in the context of a wider discussion on the psychological impacts of XLH. While some reported having considered the risk of passing on the XLH gene prior to having children, others did not mention going through a deliberate decision-making process. Among those who were concerned about passing on the gene, some delayed having children or decided not to have children, while others had considered adoption, tried IVF with donor eggs or undergone Pre-implantation Genetic Diagnosis (PGD) IVF. Feelings of guilt and being responsible for passing down the condition were commonly reported. However, a few participants stated that XLH had not affected their decision to have children because they believed the condition could be managed well.

## Discussion

The present study provided an in-depth understanding of the XLH patient experience of pain, stiffness and fatigue as well as the impact of these symptoms on patients’ physical, psychological and social well-being. For many, pain was the most salient symptom of XLH in adulthood and was commonly reported to impact on physical functioning as well as being a psychological burden. Stiffness affected participants’ agility and mobility, impacting self-care and other daily activities that require joint flexibility and fast movements. While participants often could not unambiguously attribute fatigue symptoms to XLH, most described it as having a physical quality characterised by a lack of energy. Few had coping mechanisms other than rest. For most, the impact of fatigue was limited to needing to take more rest and reduced social activity, although more severely affected participants also reported major impacts on work and housework.

The study also gave novel insights into the psychosocial impact of XLH as a lifelong hereditary condition. Concern about the future in the context of worsening symptoms over time and feelings of guilt over passing on the XLH gene to children were common.

The study also confirmed the symptoms and impacts described by Theodore-Oklota and colleagues among a more heterogenous sample across a number of European countries [11]. The results highlighted the relevance of skeletal, stiffness, pain, fatigue and dental symptoms as described by Theodore-Oklota and colleagues. Similarly, impacts on physical functioning, and the resulting impacts on ADLs, psychological well-being, sleep and the use of assistive devices and adaptations were also confirmed.

The results of the present study show that adults with XLH suffer symptoms which impact on their mobility, ADLs and HRQoL despite the fact that their skeletons have stopped growing. Symptoms often do not appear responsive to vitamin D and phosphate therapy and are reported to progress as patients age. Many of these symptoms may be due to progressive degenerative joint disease secondary to the effects of childhood rickets, since adults with XLH often require early joint replacement and other orthopaedic procedures [14]. Other symptoms, however, could be due to the continuing effects of elevated FGF23.

Pain is a salient symptom in many musculoskeletal diseases, including osteoarthritis, a common co-morbid condition of XLH. It limits physical functioning, impacting on a wide range of ADLs and psychological well-being in osteoarthritis and XLH patients alike [15, 16]. However, osteoarthritis is typically localised (e.g. knees or hips) and manifests later in life, whereas XLH is a lifelong progressive condition affecting overall bone health that results in skeletal deformities and short stature in addition to poor physical functioning. XLH patients may experience an additional psychological burden due to their physical appearance and the hereditary and progressive nature of their condition, although the commonly reported mental resilience resulting from XLH might reduce the perceived impact of their condition.

Clinical trials in XLH have used patient-reported outcome (PRO) instruments developed for more common musculoskeletal or unrelated conditions to measure pain, stiffness and fatigue in XLH patients. The present study results confirm the importance and impact of these symptoms on everyday life in XLH and therefore highlight the importance of measuring these symptoms in XLH trials. Furthermore, although developing condition-specific measures in a heterogeneous rare disease is challenging, the presented data helps to highlight important issues which any PRO used in XLH should measure.

### Study limitations

The study results should be interpreted in light of several research limitations. Generalisability of the study results is limited by the fact that the sample recruited through XLH patient associations may not be representative of the wider XLH patient population. While patients residing in five European countries were reached, a large proportion of the sample were from the United Kingdom. We could not confirm if there were meaningful between-country differences due to the small sample sizes and heterogeneity in patient experiences within countries. However, given the nature of XLH, there were no a priori hypotheses to assume that the patient experience would be systematically different between countries. To reduce sample bias, interviews were conducted over the telephone, imposing no geographical restrictions on participation and thus facilitating access to a larger proportion of the XLH population than would have been possible with face-to-face interviews. The sample had a relatively high proportion of participants with an educational qualification of degree level or above, suggesting the sample may have been more highly educated than the wider XLH patient population. A relatively high proportion within the sample were female; however, as XLH is associated with a defective gene on the X chromosome, the prevalence of XLH is expected to be higher among women. Furthermore, all results are based on patient self-report and are not benchmarked against clinical indicators or other assessments of patient health.

## Conclusion

This study is the first to provide an in-depth understanding of the adult XLH patient experience of pain, stiffness and fatigue symptoms, their impact on HRQoL and the interrelatedness of these symptoms. The psychosocial impact of XLH was also explored in detail to understand the experience of living with a hereditary, lifelong progressive disease.

## Electronic supplementary material

Below is the link to the electronic supplementary material.

Supplementary file1 (DOCX 17 kb)

Supplementary file2 (DOCX 36 kb)

Supplementary file3 (DOCX 24 kb)

## References

[1] Carpenter T, Imel E, Holm I, Jan de Beur S, Insogna K (2011). A clinician’s guide to X-linked hypophosphatemia. Journal of Bone and Mineral Research.

[2] Pavone V, Testa G, Iachino SG (2015). Hypophosphatemic ricketsL etiology, clinical features and treatment. European Journal of Orthopaedic Surgery and Traumatology.

[3] Linglart A, Biosse-Duplan M, Briot K (2014). Therapeutic management of hypophosphatemic rickets from infancy to adulthood. Endocrine Connections.

[4] Forestier-Zhang L, Watts L, Turner A (2016). Health-related quality of life and a cost-utility simulation of adults in the UK with osteogenesis imperfecta, X-linked hypophosphatemia and fibrous dysplasia. Orphanet Journal of Rare Diseases.

[5] Reid I, Hardy D, Murphy W (1989). X-linked hypophosphatemia: A clinical, biochemical, and histopathologic assessment of morbidity in adults. Medicine (Baltimore).

[6] Beck-Nielsen S, Brusgaard K, Rasmussen L (2010). Phenotype presentation of hypophosphatemic rickets in adults. Calcified Tissue International..

[7] Hanisch M, Bohner L, Sabandal M, Kleinheinz J, Jung S (2019). Oral symptoms and oral health-related quality of life of individuals with x-linked hypophosphatemia. Head and Face Medicine.

[8] James M, Roudsari R (2019). Prosthetic rehabilitation of a patient with X-linked hypophosphatemia using dental implants: A case report and review of the literature. International Journal of Implant Dentistry.

[9] Che H, Roux C, Etcheto A (2016). Impaired quality of life in adults with X-linked hypophosphatemia and skeletal symptoms. European Journal of Endocrinology.

[10] Skrinar A, Dvorak-Ewell M, Evins A (2019). The lifelong impact of X-linked hypophosphatemia: Results from a burden of disease survey. Journal of Endocrine Society.

[11] Theodore-Oklota C, Bonner N, Spencer H, Arbuckle R (2018). Qualitative research to explore the patient experience of X-linked hypophosphatemia and evaluate the suitability of the BPI-SF and WOMAC as clinical trial end points. Value in Health.

[12] Braun V, Clarke V (2006). Using thematic analysis in psychology. Qualitative Research in Psychology.

[13] Braun V, Clarke V (2014). What can “thematic analysis” offer health and wellbeing researchers?. International Journal of Qualitative Studies on Health and Well-being.

[14] Chesher D, Oddy M, Darbar U (2018). Outcome of adult patients with X-linked hypophosphatemia caused by PHEX gene mutations. Journal of Inherited Metabolic Disease.

[15] Nyvang J, Hedstrom M, Gleissman SA (2016). It’s not just a knee, but a whole life: A qualitative descriptive study on patients’ experiences of living with knee osteoarthritis and their expectations for knee arthroplasty. International Journal of Qualitative Studies on Health and Well-being.

[16] Stamm T, Pieber K, Crevenna R, Dorner T (2016). Impairment in the activities of daily living in older adults with and without osteoporosis, osteoarthritis and chronic back pain: A secondary analysis of population-based health survey data. BMC Musculoskeletal Disorders.

