# Supplementary material for: Exploring the burden of X-linked hypophosphatemia: a European multi-country qualitative study
Source: Qual Life Res. 2020 Mar 11;29(7):1883–93. doi: 10.1007/s11136-020-02465-x (PMC7295835; doi:10.1007/s11136-020-02465-x)
Supplement: Supplementary file 1 — Supplementary file1 (DOCX 17 kb) [file 11136_2020_2465_MOESM1_ESM.docx]

# Background form: Adult XLH patient

1. How old were you when you were first diagnosed with X linked hypophosphatemia (XLH)? |_|_|
   - Don’t know
2. How severe would you say is your XLH currently?

- Mild
- Moderate
- Severe
- Don’t know

1. Which treatments do you currently receive for your XLH? *Please tick all that apply*

- Phosphate
- Calcitriol / activated vitamin D
- Vitamin D
- Surgery
- Dental procedures
- Physiotherapy
- Other, please specify: ___
- Don’t know

1. Which treatments have you received in the past for your XLH? *Please tick all that apply*

- Phosphate
- Calcitriol / activated vitamin D
- Vitamin D
- Growth hormone
- Surgery
- Dental procedures
- Physiotherapy
- Other, please specify: ___
- Don’t know

1. Do any other members of your family have XLH? *Please tick all that apply*
   - Mother
   - Father
   - Brother or sister
   - Other, please specify: ___
2. Do you have any other illnesses or conditions which limit your daily activities?
   - Yes, please specify: ___
   - No
3. What is your age? |_|_|
4. Are you…
   - Male
   - Female
   - Other
   - Prefer not to answer
5. What is your current living situation? *Select one*

- Living with partner / spouse
- Living alone
- Living with relative(s)
- House sharing
- Other
- Prefer not to answer

1. Which of the following best describes your current employment status? *Select one for each column*

- Employed / Self-employed, full-time
- Employed / Self-employed, part-time
- Homemaker
- Unemployed, seeking work
- Unable to work due to health issues
- Retired
- Student
- Other
- Prefer not to answer

1. Which of the following best describes your highest level of educational qualification? *Select one*

- No formal qualifications
- Primary education
- Secondary education
- Degree level or higher
- Other
- Prefer not to answer
