# Supplementary material for: Exploring the burden of X-linked hypophosphatemia: a European multi-country qualitative study
Source: Qual Life Res. 2020 Mar 11;29(7):1883–93. doi: 10.1007/s11136-020-02465-x (PMC7295835; doi:10.1007/s11136-020-02465-x)
Supplement: Supplementary file 2 — Supplementary file2 (DOCX 36 kb) [file 11136_2020_2465_MOESM2_ESM.docx]

# INTERVIEW GUIDE: XLH ADULTS

# Introduction

Good (morning/afternoon/evening), introduce self.

Thank you for taking the time for today’s interview. The purpose of this study is to understand the impact of X linked hypophosphatemia (XLH) on people’s lives. I am interested in hearing about your experiences with the disease, its impact on your quality of life.

Our conversation will be recorded so that we can accurately represent what you are saying during the discussion in our research report. We will now begin recording. OK?

*(If participant does not wish to be recorded, interviewer should not proceed with the interview)*

**START RECORDING**

***Begin Recorder:*** Today is [date]. This is participant ID [Insert number here]

- Do I have your permission to record this interview? [YES/NO]
- Please could you confirm that you understand that everything you tell us today will be kept confidential and no one will be able to find out what you personally said in our reports? [YES/NO]
- Are you happy for us to start with the interview? [YES/NO]

I would like to clarify a few points before we begin:

- I am not a medical doctor, so I am not qualified to give medical advice. If you have any questions about your condition as a result of our conversation today I advise you to follow up with your regular doctor.
- There are no right or wrong answers, we understand that everyone has different experiences and are interested in what you have to say.
- Please speak loudly enough to be heard for the recording.
- Do you have any questions before we begin? If yes, please ask the participant to elaborate.

**[MODERATOR NOTE 1: throughout interview, only use probes where appropriate, taking patient profile and what has previously been discussed into account]**

**[MODERATOR NOTE 2: throughout adult interviews, probe for pain, fatigue and stiffness experienced and impact of pain and fatigue on HRQL and daily life/activities where possible]**

# Background

First, I would like to get an idea about who you are and your life.

- Please could you tell us about yourself?

**[PROBE: age, living situation: e.g. alone/with partner/family/housemates, college/work, caregiver status: caring for children/older family members]**

- Could you tell me about any interests or hobbies you have?
- Moving on to your XLH, how did you first get diagnosed with XLH?

**[PROBE: symptoms, time from presentation to diagnosis, who, what, where, patient pathway to diagnosis, family history]**

- If you can remember, what was it like when you were first diagnosed with XLH?

**[PROBE: time/journey to diagnosis, initial response, emotions, impact on future outlook]**

- Do you currently experience any symptoms due to XLH?

**[PROBE:**

1. **Musculoskeletal: stiffness/difficulty bending limbs or joints, short stature, bone bowing, bone weakness/soft bones/fractures/osteomalacia, osteoarthritis**
2. **Pain: joint pain, bone pain, muscle pain, dental pain**
3. **Fatigue: fatigue/tiredness, tiredness, weakness, low energy, sleepiness**
4. **Dental: dental abscesses, dental deformities**
5. **Sensory: loss of balance/vertigo, loss of hearing, tinnitus, vision loss**
6. **Other clinical conditions: cardiometabolic/metabolic syndrome]**

# Daily life

I would now like to try to understand how living with XLH impacts your life.

- Can you talk me through a typical day for you, thinking about your daily routines and activities whether on a weekday or a weekend/holiday and how XLH affects or does not affect the things you do?

**[PROBE: getting up, washing, dressing, choice of clothes, breakfast, going to college/work, time spent at college/work, social activities, leisure activities/hobbies, sports/exercise, relationships/family/friendships, dinner, evening routine, how well you sleep]**

- What are the main ways XLH impacts on your daily life?

**[PROBES:**

1. **Daily activities: work, housework, caregiving if relevant, getting dressed, shopping, laundry, getting in/out of car, getting in/out of bath or shower**
2. **Physical functioning: difficulty walking, sitting, standing, bending, range of motion, arising, with stairs, sport/exercise**
3. **Use of assistive devices and adaptations: modified home and equipment, walking aids**
4. **Emotional/psychological: depression/low mood, frustration, anxiety/worries, confidence/self-esteem, impact on identity, passing gene on to children**
5. **Cognition: concentration, clarity/speed of thinking, difficulty planning/making decisions/finishing tasks**
6. **Sleep/fatigue: waking in the night (e.g. due to pain), falling asleep, duration of sleep, fatigue during the day**
7. **Social impact: relationships, family, school, friendships, strangers, disclosure to friends and strangers**

**For each impact probe *why* XLH has this impact on them]**

- How does the way XLH impacts on your life make you feel?

**[PROBE: type of emotion]**

- Can you tell me about anything you do to cope with the impact of XLH on your life?

**[PROBE: treatment/medication, support, planning, avoiding certain activities, rest]**

- 1. How much time/effort does this take?
  2. How does this make you feel?

# Treatment and Care

- You said that you have received a) [treatment]; b) [surgery]; c) other therapy e.g. physiotherapy. How does it help/has it helped you cope with XLH?

**[PROBE: phosphate, vitamin D: activated (e.g. Calcitriol) or plain, growth hormone, type of surgery, dental procedures, physiotherapy]**

**[PROBE2: when did participant receive each treatment? Still receiving treatment or past treatment when still a minor? If stopped taking treatment, why?]**

- Are there any downsides or limitations to receiving/using [treatment/surgery/other therapy]?

**[PROBE:**

1. **side effects in general, gastrointestinal side effects such as diarrhoea and abdominal pain in particular;**
2. **limited effectiveness in general, more specifically issues unresolved by treatment: e.g. stature, disturbed gait, mobility issues, fractures, pain in legs, back pain, stiffness in joints, fatigue, psychological and social impact;**
3. **impact of treatment on quality of life in general, pill/treatment burden, hospital appointments, medical tests, surgery and rehabilitation in particular]**
4. **Emotional/psychological impact of treatment – embarrassed, anxious, guilty, etc.]**

- Have you ever skipped doses or taken a break from your medication/treatment?
- **[IF YES]** Why?
- **[IF YES]** Did you re-start treatment? Why (not)?
- Do you need/use reminders? Do other people help remind you to take medication?
- How would you say you cope with your XLH?

**[PROBE: very badly/quite badly/okay/quite well/very well]**

**[PROBE2: which aspects of XLH make you respond in this way?]**

- Could you tell me about the care and support you receive from healthcare professionals, e.g. doctors and nurses?

**[PROBE: type of support, frequency and duration]**

- Could you tell me whether, and if so, what support you receive from family and friends?

**[PROBE: type of support, frequency and duration]**

# Pain

**[IF PAIN REPORTED]** You mentioned that you experienced pain due to your XLH.

- Where do you experience pain?

**[PROBES: legs, arms, hands, hips, back, shoulder, neck]**

- How would you describe the pain you experience in your own words?

**[PROBES: joint vs. bone vs. muscle or unsure; aching, burning/hot, cold, dull, gnawing, heavy, nagging, numb, penetrating, sharp, stabbing, shooting, tender, tight/cramping, tingling, throbbing, radiating, squeezing, deep vs. surface pain]**

- How severe is the pain a) on average; b) at its worst; c) at its best?

**[PROBES: none/mild/moderate/severe/extreme]**

- How unpleasant would you say is the pain you experience?

**[PROBES: not unpleasant/slightly unpleasant/moderately unpleasant/very unpleasant/extremely unpleasant]**

- How often do you have pain?

**[PROBES: persistent vs. fluctuating, with or without pain-free periods,**

**every minute/hour/day/week/month]**

- When you have pain, how long does it last?

**[PROBES: intermittent vs. continuous; seconds/minutes/hours/days]**

- When did you first start experiencing pain? Have things changed over time?
- **[PROBES: childhood/adolescence/young adulthood/recently]**

**[MODERATOR NOTE: do not probe further if participant does not remember]**

- Are there specific times that you experience more or less pain?

**[PROBES: morning/afternoon/evening/night, time of year]**

- Are there specific triggers that provoke or increase your pain?

**[PROBES: pressure/touch/brushing/types of movement]**

- Do you know what causes the pain?

**[PROBES: neuropathic pain vs. bone pain or unsure; association with physical deformities]**

- Is there anything you do to reduce the pain? Do you take medication to reduce pain?

**[PROBES: pain killers: anti-inflammatories, opioids; phosphate]**

- **[IF TAKING PAIN MEDICATION]** To what extent does it help reduce the pain?
- How does the pain impact your daily life?

**[PROBES:**

1. **Daily activities: work, housework, caregiving if relevant, getting dressed, shopping, laundry, getting in/out of car, getting in/out of bath or shower**
2. **Physical functioning: difficulty walking, sitting, standing, bending, range of motion, arising, with stairs, sport/exercise**
3. **Use of assistive devices and adaptations: modified home and equipment, walking aids**
4. **Emotional/psychological: depression/low mood, frustration/irritability, anxiety, confidence/self-esteem**
5. **Mental: memory, concentration**
6. **Sleep: waking in the night (due to pain), falling asleep, insomnia**
7. **Social impact: family, school, friendships, strangers**

**For each impact probe *why* pain has this impact on them]**

# Stiffness

**[IF STIFFNESS REPORTED]** You mentioned that you experienced stiffness due to your XLH.

- Where do you experience stiffness?

**[PROBES: legs, arms, hands, hips, back, shoulder, neck]**

- How would you describe the stiffness in your own words?
- How severe is the stiffness a) on average; b) at its worst; c) at its best?

**[PROBES: mild/moderate/severe/extreme]**

- How often do you experience stiffness?

**[PROBES: regular vs. variable, every minute/hour/day/week/month]**

- When you have stiffness, how long does it last?

**[PROBES: intermittent vs. continuous; seconds/minutes/hours/days]**

- When did you first start experiencing stiffness? Have things changed over time?
- **[PROBES: childhood/adolescence/young adulthood/recently]**
- **[MODERATOR NOTE: do not probe further if participant does not remember]**
- Are there specific times when/situations in which you experience more or less stiffness?

**[PROBES: seasons, morning/afternoon/evening/night, after a period of rest/not moving, other health and life circumstances,]**

- Is there anything you do to reduce the stiffness?

**[PROBES: massage/physio/treatment]**

To what extent does it help?

- How does the stiffness impact your daily life?

**[PROBES:**

1. **Daily activities: work, housework, caregiving if relevant, getting dressed, shopping, laundry, getting in/out of car, getting in/out of bath or shower**
2. **Physical functioning: difficulty walking, sitting, standing, bending, range of motion, arising, with stairs, sport/exercise**
3. **Use of assistive devices and adaptations: modified home and equipment, walking aids**
4. **Emotional/psychological: depression/low mood, frustration/irritability, anxiety, confidence/self-esteem**
5. **Mental: e.g. memory, concentration**
6. **Sleep: waking in the night (due to stiffness), falling asleep, insomnia**
7. **Social impact: family, school, friendships, strangers**

**For each impact probe *why* stiffness has this impact on them]**

# Fatigue

**[IF FATIGUE REPORTED]** You mentioned that you experienced fatigue/tiredness due to your XLH.

- How would you describe the fatigue you experience in your own words?

**[PROBES: low energy, drained, drowsiness, exhaustion, feeling worn out/run-down, not rested, sluggish, weariness]**

- How severe is the fatigue/tiredness?

**[PROBES: mild/moderate/severe/extreme]**

- How often do you experience fatigue/feel tired?

**[PROBES: regular vs. variable; every minute/hour/day/week/month]**

- When you experience fatigue/tiredness, how long does it last?

**[PROBES: continuous vs. intermittent; seconds/minutes/hours/days]**

- When did you first start experiencing fatigue?

**[PROBES: childhood/adolescence/young adulthood/recently]**

**[MODERATOR NOTE: do not probe further if participant does not remember]**

- Are there specific times that you experience more or less fatigue/tiredness?

**[PROBES: morning/afternoon/evening/night, time of year, other health and life circumstances]**

- Do you know what causes the fatigue/tiredness or makes it worse?

**[PROBES: lack of sleep; effort needed to complete daily activities, exercise]**

- Is there anything you do or take to reduce the fatigue/tiredness?

**[PROBES: rest, caffeine, dietary supplements, exercise]**

To what extent does it help?

- How does the fatigue/tiredness impact your daily life?

**[PROBES:**

1. **Daily activities: work, housework, caregiving if relevant, getting dressed, shopping, laundry, getting in/out of car, getting in/out of bath or shower, watching television**
2. **Physical functioning: difficulty walking, sitting, standing, bending, range of motion, arising, with stairs, sport/exercise**
3. **Use of assistive devices and adaptations: modified home and equipment, walking aids**
4. **Emotional/psychological: bothered/distressed about fatigue, depression/low mood, frustration, anxiety, confidence/self-esteem, motivation to do things, enjoyment of life/happiness**
5. **Cognitive: concentration, clarity/speed of thinking, difficulty planning/making decisions/finishing tasks**
6. **Sleep: sleeping in daytime/early in the evenings/late in the mornings**
7. **Social impact: family, school, friendships, strangers**

**For each impact probe *why* fatigue has this impact on them]**

# Unmet Needs and Future Treatment

- Would you say you have any needs as a person with XLH that are not met through the treatments, healthcare services and support you receive?
- Thinking of the past and current treatments you have received for XLH, how would you like new and future treatments to improve on existing ones?

**[PROBE: cure, effectiveness in general, reduce pain caused by XLH in particular, fewer side effects, lower burden of administration/surgical procedures]**

- How would better treatment options impact on your life as a person with XLH?

**[PROBE: mobility, self-care, usual activities, pain, mental health; options and outlook in life; any impacts previously mentioned]**

# Conclusion

We are soon coming to the end of this interview.

- To conclude, how would you summarise the impact of XLH on your life, both positive and negative?
- Are there any other ways in which your quality of life is impacted by XLH that we have not discussed?

Thank you for your help with this research.

We really appreciate the time you have taken to participate in this study.
