# Supplementary material for: Exploring the burden of X-linked hypophosphatemia: a European multi-country qualitative study
Source: Qual Life Res. 2020 Mar 11;29(7):1883–93. doi: 10.1007/s11136-020-02465-x (PMC7295835; doi:10.1007/s11136-020-02465-x)
Supplement: Supplementary file 3 — Supplementary file3 (DOCX 24 kb) [file 11136_2020_2465_MOESM3_ESM.docx]

# CODE FRAME: XLH ADULTS

## General codes

**SYMPTOMS**

- Mobility
- Skeletal
- Pain, general
- Bone pain
- Joint pain
- Muscle pain
- Dental pain
- Stiffness, general
- Stiff sensation
- Stiffness restricting mobility
- Fatigue, general
- Mental fatigue
- Physical fatigue
- Dental, general
- Dental abscesses
- Balance
- Hearing
- Tinnitus
- Vision

**IMPACT**

- Physical appearance
- Self-care
- Financial
- Children with XLH
- Reliance on other people
- Daily activities
- Physical functioning
- Time and effort
- Stigma/bullying/lack of understanding
- Psychological/emotional
- Cognition
- Social
- Sleep

**FEELINGS AND SENTIMENTS**

- Embarrassed
- Getting on with it
- Part of identity
- Acceptance
- Angry/frustrated
- Concerned/worried/scared
- Drained
- Guilty
- Sad
- Restricted
- Low confidence
- Character building
- Negative, other
- Positive, other

**COPING**

- HCP care and support
- Coping strategies
- Assistive devices/adaptations
- Family and friends support
- Unmet needs

**TREATMENT EXPERIENCE**

- Inconvenience
- Symptom alleviation
- Effectiveness
- Side effects
- Medication adherence
- Impact on daily life
- Emotional impact of treatment

**NEW AND FUTURE TREATMENTS**

- Burosumab
- New and future treatment

## Pain-specific codes

- Location
- Qualitative sensation
- Severity
- Frequency
- Fluctuations
- Duration
- Onset
- Changes over time
- Time of day
- Time of year
- Triggers
- Cause
- Alleviation

## Stiffness-specific codes

- Location
- Qualitative sensation
- Severity
- Frequency
- Fluctuations
- Duration
- Onset
- Changes over time
- Time of day
- Time of year
- Triggers
- Cause
- Alleviation

## Fatigue-specific codes

- Qualitative sensation
- Severity
- Frequency
- Fluctuations
- Duration
- Onset
- Changes over time
- Time of day
- Time of year
- Triggers
- Cause
- Alleviation
